# Size-Dependent Interplay of Volume Exclusion Versus Soft Interactions: Cytochrome *c* in Macromolecular Crowded Environment

**DOI:** 10.3389/fmolb.2022.849683

**Published:** 2022-05-25

**Authors:** Zahoor Ahmad Parray, Faizan Ahmad, Anis Ahmad Chaudhary, Hassan Ahmad Rudayni, Mohammed Al-Zharani, Md. Imtaiyaz Hassan, Asimul Islam

**Affiliations:** ^1^ Centre for Interdisciplinary Research in Basic Sciences, Jamia Millia Islamia, New Delhi, India; ^2^ Department of Chemistry, Indian Institute of Technology Delhi, New Delhi, India; ^3^ Department of Biochemistry, School of Chemical and Life Sciences, Jamia Hamdard, New Delhi, India; ^4^ Department of Biology, College of Science, Imam Mohammad Ibn Saud Islamic University (IMSIU), Riyadh, Saudi Arabia

**Keywords:** protein folding, cytochrome c, macromolecular crowding, isothermal titration calorimetry, molecular docking

## Abstract

Even though there are a great number of possible conformational states, how a protein generated as a linear unfolded polypeptide efficiently folds into its physiologically active form remained a fascinating and unanswered enigma inside crowded conditions of cells. In this study, various spectroscopic techniques have been exploited to know and understand the effect and mechanism of action of two different sizes of polyethylene glycols, or PEGs (molecular mass ∼10 and ∼20 kilo Daltons, kDa), on cytochrome *c* (cyt *c*). The outcomes showed that small size of the PEG leads to perturbation of the protein structure, and conversely, large size of the PEG has stabilizing effect on cyt *c*. Moreover, binding measurements showed that small size of PEG interacts strongly *via* soft interactions compared to the larger size of PEG, the latter being governed more by excluded volume effect or preferential exclusion from the protein. Overall, this finding suggests that conformations of protein may be influenced in cellular crowded conditions *via* interactions which depend upon the size of molecule in the environment. This study proposes that both volume exclusion and soft (chemical) interactions governs the protein’s conformation and functional activities. The cellular environment’s internal architecture as evident from crowder size and shape in this study has a significant role.

## Graphical Abstract

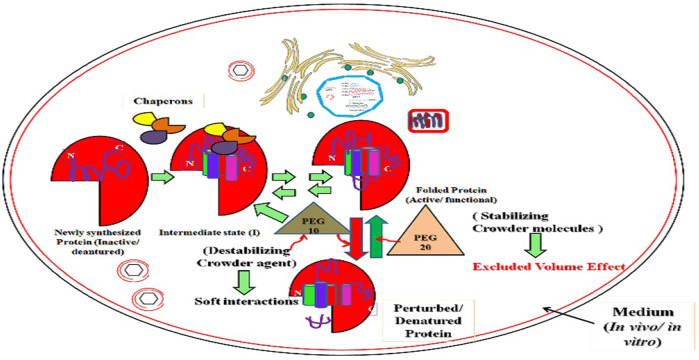

## Highlights

➢ Macromolecular crowding in cells has a considerable effect on the structure of proteins, which may affect its biological activity.
➢ Size-dependent crowding effect can be used as an approach to discern the effects on proteins in cellular conditions.
➢ Exclusion volume effect favors stabilization by large crowding agents; conversely, soft interactions favor destabilization by small crowding agents.

## 1 Introduction

Various studies on protein folding in crowded systems with a range of parameters (size, shape, and concentrations) were carried out to understand the phenomenon (Van Den Berg et al., 1999; Van Den Berg et al., 2000; Galan et al., 2001; Zhou et al., 2004; Parray et al., 2019; Parray et al., 2020a; Parray et al., 2020b; Parray et al., 2020c; Parray et al., 2020d; Nasreen et al., 2020). Protein folding has been examined in the presence of macromolecular crowders like polyethylene glycols (PEGs), ficolls, dextran, and others under physiological environments (Sasahara et al., 2003; Rawat et al., 2010; Qu et al., 2012; Zhang et al., 2012; Sarkar et al., 2013a; Kundu et al., 2015; Shahid et al., 2015; Parray et al., 2017; Shahid et al., 2017; Nasreen et al., 2018; Parray et al., 2019; Parray et al., 2020a; Parray et al., 2020b; Parray et al., 2020c; Parray et al., 2020d; Nasreen et al., 2020). It is known that macromolecular crowding in cells has a massive effect on biological activity, but predictive studies are relatively unexplored when it comes to its implications. The proteins in such crowded conditions are surrounded by various other molecules (small and large sized) which results in the structural change in conformation of proteins. The cellular environment is complex, heterogeneous, and highly crowded (Goodsell, 2012; Weiss, 2014); the *in vitro* environment is significantly distinctive to execute majority of the biophysical studies (Parray et al., 2017; Parray et al., 2020a; Nasreen et al., 2020). Due to the overcrowded milieu in concentrated solution, roughly a third of the volume accessible to macromolecules is excluded. Two aspects of excluded volume are soft interactions and steric repulsions or hard interactions (Sarkar et al., 2013a). Hard-core interactions include attraction as well as repulsions where soft interactions can be subtracted and/or added. It is easy to understand how repulsive strong interaction enhances the excluded volume (Minton, 1981; Sarkar et al., 2013b). Repulsion is said to be increased when the test protein and the crowder molecule have an identical charge. As a result, repulsive interactions lead to an increase in the excluded volume, where the greater size of the crowder molecule plays a key role (Minton, 1981; Shahid et al., 2017; Das and Sen, 2019). Soft interactions can be attractive or repulsive and, thus, structurally constructive or destructive in that order (Sarkar et al., 2013b; Parray et al., 2019). As a result, chemical (soft) interactions, which are contrary to the hardcore repulsions (generally, they stabilize proteins), may destabilize or stabilize proteins. The excluded volume effect is a valuable notion for understanding the effects of crowding on proteins because of the dependencies of soft and hard interactions (Shahid et al., 2015; Shahid et al., 2017; Parray et al., 2019; Parray et al., 2020d). Proteins are heteropolymers with the unique propensity to fold quickly into compact and specified conformations. Protein interiors are tightly packed with low empty volumes (Chothia, 1975), revealing particular inter-residue interactions that govern secondary and tertiary structure. Compactness thus is a significant aspect of folded protein conformations; nevertheless, the dynamics of compaction during protein folding from long random-coil structures are still poorly understood (Chothia, 1975).

Under physiological conditions, our group has recently characterized two distinct intermediate states in myoglobin (Mb) induced by two different sizes of PEG at higher concentrations: molten globule (MG) by PEG 10 kDa (Parray et al., 2017) and pre-molten globule (PMG) by PEG 400 Da (Parray et al., 2019) under physiological conditions. We recently conducted a study in which we found that PEG 400 and ethylene glycol (EG) have different impacts on the tertiary structure of cyt *c* (Parray et al., 2020a; Parray et al., 2020c). However, the secondary structure of the protein was not disrupted by PEG 400, which leads to the formation of an MG structure at high concentration (300 mg ml^−1^) (Parray et al., 2020a), while EG has stabilizing effects on the structure of the protein (Parray et al., 2020c). It was proposed that protein-solvent preferential interaction or/and kosmotropic effect of EG leads to the protein compaction (Parray et al., 2020c). In addition, PEG 4 kDa was observed to have noteworthy effect on the tertiary and secondary structure of Mb and cyt *c*, where significance of concentration in the crowded system was elucidated (Parray et al., 2021a; Parray et al., 2021c).

The folding dynamics of cytochrome *c* (cyt *c*), a globular protein of 104 residues, is intensively exploited as experimental research (Akiyama et al., 2002) to visualize the folding pathway of cyt *c.* In order to understand the mechanism of protein folding in the crowded system, we established a size-dependent crowded environment to mimic cellular systems where proteins are exposed to various forces and face conformational changes during the folding–unfolding process influenced by a range of molecules (small, intermediate, and large sized) (Ellis, 2001; Ellis, 2007; Chen et al., 2008; Goodsell, 2012).

In this study, we have chosen two different sizes of PEG in which one is twofold of the other (PEG 10 and PEG 20 kDa) to know the size-dependent crowded effect on the structure of the protein (cyt *c*) using various spectroscopic approaches. A comparative analysis was prepared to know the impact of size-dependent changes on the protein in comparable circumstances (pH 7.0 and 25°C). Moreover, interaction studies were carried out to know how crowders of different sizes influence and what type of interactions they create with the protein. The effects of volume exclusion on the free energy of macromolecules in crowded and confined systems, as well as the consequences of crowding, and confinement on structural stability and intra-molecular interactions are addressed here. Overall, this finding suggests that proteins can be influenced in cellular crowded conditions *via* distinct routes and interactions, depending upon the size of surrounding molecules.

## 2 Materials and Methods

### 2.1 Materials

Lyophilized form of horse heart cytochrome *c* and PEGs (10 and 20 kDa) were ordered from Sigma chemical company (United States). Himedia (Germany) provided sodium phosphate monobasic and di-sodium hydrogen phosphate anhydrous. Merck Millipore Ltd. (Cork) provided the filters, which had a pore size of 0.22 μm.

### 2.2 Methods

#### 2.2.1 Preparation of Protein and Reagents

As previously described (Parray et al., 2020a; Parray et al., 2020c), the protein solution (75 mg ml^−1^ of lyophilized powdered cyt *c*) was prepared in the 50 mM phosphate buffer and was oxidized with potassium ferricyanide (Goto et al., 1990). Subsequently, the mixture was dialyzed three times against a 50 mM phosphate buffer at 4°C to eliminate any excess salts and potassium ferricyanide. The dialysis was followed by filtration of the protein using a 0.22-μm Millipore filter. The Beer–Lamberts law (*c* = *A*_410_/*εl*) was used to determine the concentration of protein, where *c* is the protein concentration in molar (M), *l* signifies cell path length in centimeter (cm), *A* is the absorbance value at 410 nm, and *ε* is the molar absorption coefficient at 410 nm (*ε*_410_, M^−1^ cm^−1^); the reported value of *ε*_410_ is 106,100 M^−1^ cm^−1^ (Margoliash and Frohwirt, 1959).

In phosphate buffer, the required amount of denaturant (guanidinium chloride, GdmCl) and PEGs (10 and 20 kDa) were dissolved and then filtered through Whatman filter paper No. 1. Concentrations of these solutions were determined using refractive index values (Nozaki, 1972; Tumolo et al., 2004). To perform optical measurements, each sample of various crowder concentrations prepared in the degassed buffer was incubated for 12–15 h (maximum time for the response of ligand–protein interaction). All measurements were made in triplicate.

#### 2.2.2 Spectroscopic Techniques

##### 2.2.2.1 UV-Visible Spectra Measurements

A Jasco V-660 UV-vis spectrophotometer attached to a Peltier-type temperature controller (ETCS761) was used for spectral measurements. For these measurements, 6–7 µM protein in a cuvette of 1.0 cm path length was used (Parray et al., 2020a; Parray et al., 2020c).

##### 2.2.2.2 Circular Dichroism Measurements

The circular dichroism (CD) spectral measurements were conducted using a Jasco Spectropolarimeter (J-1500 model) with an in-built temperature controller which attached to an external bath (MCB-100) for circulating water at 20°C. The protein concentration used was 16 µM. Cuvettes with path lengths of 1.0 and 0.1 cm were used for the near- and far-UV CD spectra measurements, respectively. The measurements of Soret CD were taken in the regions of 370–450 nm in the cuvette with 1.0 cm path length. D-10 camphor sulphonic acid was used to standardize the CD machine. For each sample, several accumulations were applied along with a baseline in order to improve the signal-to-noise ratio. The CD machine was purged with nitrogen gas at a rate of 4, 5 L per minute to remove air. The observed CD signal (mdeg) was changed to concentration-independent parameters, namely, the mean residue ellipiticity (MRE) designated as [*θ*]_λ_ (deg cm^2^ dmol^−1^), using the following equation:

$${\left[\theta \right]}_{\lambda }=M_{0}\theta_{\lambda }/10lc$$ (1)

where *θ*_λ_ is ellipticity at wavelength λ in millidegrees, *M*_0_ is the mean residue weight of the protein, *c* is the protein concentration in mg ml^−1^, and *l* is the path length of the cuvette in centimeters. [*θ*]_222_ and [*θ*]_208_ signature values in the CD spectra were used to monitor progress in the protein’s secondary structure and estimate of α-helical content in the absence and presence of crowders (Morrisett et al., 1973).

It should be noted that estimation of α-helical content or secondary structure from CD measurements may involve errors, but it should be highlighted that the results are reproduceable, and error, if it is there, may be systematic. To reduce errors, better output and less noise-to-signal ratio should be maintained in measurements. Furthermore, degassing (without bubbles) and using triplicate measurements of each sample minimize the error.

##### 2.2.2.3 Dynamic Light Scattering for Particle Size Measurements

The hydrodynamic radius (*R*_h_) of cyt *c* and cyt *c*- PEGs solutions were estimated from dynamic light scattering (DLS) at pH 7.0 and 25 ± 0.1°C using a Malvern Zetasizer Nano ZS instrument. Before taking measurements, sample solutions were filtered using 0.22-μM filters; the protein concentration in each sample used was 0.5 mg ml^−1^. Each sample run had a detection angle of 12.8° and a scattering angle of 175°, as well as a 4-mW Helium–Neon laser operating at 632.8 nm with a beam diameter of 0.63 nm (1/e2). 0.5 mg ml^−1^ of cyt *c* within PEGs (PEGs 10 and 20) of concentrations of 0, 100, and 300 mg ml^−1^ were put in the standard Malvern polystyrene cuvette of 10 mm. Each sample was measured three to four times, and data analysis was made using the software Zetasizer Ver. 7.13.

#### 2.2.3 Binding Studies

##### 2.2.3.1 Isothermal Titration Calorimetry

For isothermal titration calorimetry measurements, a VP ITC Calorimeter (MicroCal, Northampton, MA) was used. The crowders (PEGs 10 and 20) were titrated into the calorimeter cell containing the protein (cyt *c*). Protein and the ligand binding was monitored at their different ratios, but 1:30 (protein:crowder) showed the best results. 10-µl aliquots of crowders were passed per injection excluding the first one, which was 5 µl. MicroCal Origin ITC software was used to normalize and analyze the data (Parray et al., 2019; Parray et al., 2020b). All measurements were taken using 50 mM phosphate buffer (pH 7.0) at 25°C (298 K). The raw data were fitted by Origin 8.0 using sequential binding models; the final fit gives the thermodynamic binding parameters such as changes in standard enthalpy (Δ*H* ^o^) and entropy (Δ*S* ^o^) and the association constant (*K*_a_). The change in standard Gibbs free energy (Δ*G* ^o^) was determined using the following equation:

$$\Delta G^{\text{o}}=-\mathrm{RT}\text{ln}K_{a}=\Delta H^{\text{°}}-T\Delta S^{\text{°}}$$ (2)

where *R* and *T* are the gas constant and absolute temperature (in Kelvin), respectively.

##### 2.2.3.2 Computational Studies (Molecular Docking Studies)

PyRx software was used to dock PEGs (10 and 20 kDa) to a macromolecule (cyt *c*) in order to find the binding site, binding affinity, and the residues participating in the interaction (Parray et al., 2020b). PyRx software is a combination of various softwares (AutoDockVina, Open Babel, AutoDock 4.2, Mayavi, etc.). PyRx uses Vina and AutoDock 4.2 that perform computational binding of ligand and receptor (Dallakyan and Olson, 2015). The input files ligand (Source Pubchem), (PEGs), and macromolecule, cyt *c* (PDB id: 1hrc) in .pdb format were changes to pdbqt files using Autodock software. The Autogrid 4 module covers all the amino acid residues of the protein. Here, the grid dimensions x, y, and z were fixed to be 89, 86, and 81 Å (receptor axis coordinates) and 0.385 Å as the grid space size for PEG 10-cyt *c,* and 80, 98, and 88 Å (receptor axis coordinates) and 0.585 Å for PEG 20-cyt *c*. Using the Lamarckian Genetic Algorithm (LGA), docking simulations were run to find the best configuration for the macromolecule and ligand. BOVIA Discovery Studio (Biovia, 2015), as well as LigPlot v.4.5.3 provided by EMBL-EBI, uses Java as a programming language (Wallace et al., 1995) and showed the computational studies of the PEGs binding to the protein to generate a 2D interaction plot. The docked pose was ultimately visualized using PyMOL (Seeliger and De Groot, 2010). The binding free energies obtained from molecular docking studies were used to calculate the binding constant values as per the earlier reported method (Parvez et al., 2019).

## 3 Results

### 3.1 UV–Visible Absorption Studies

**FIGURE 1:**
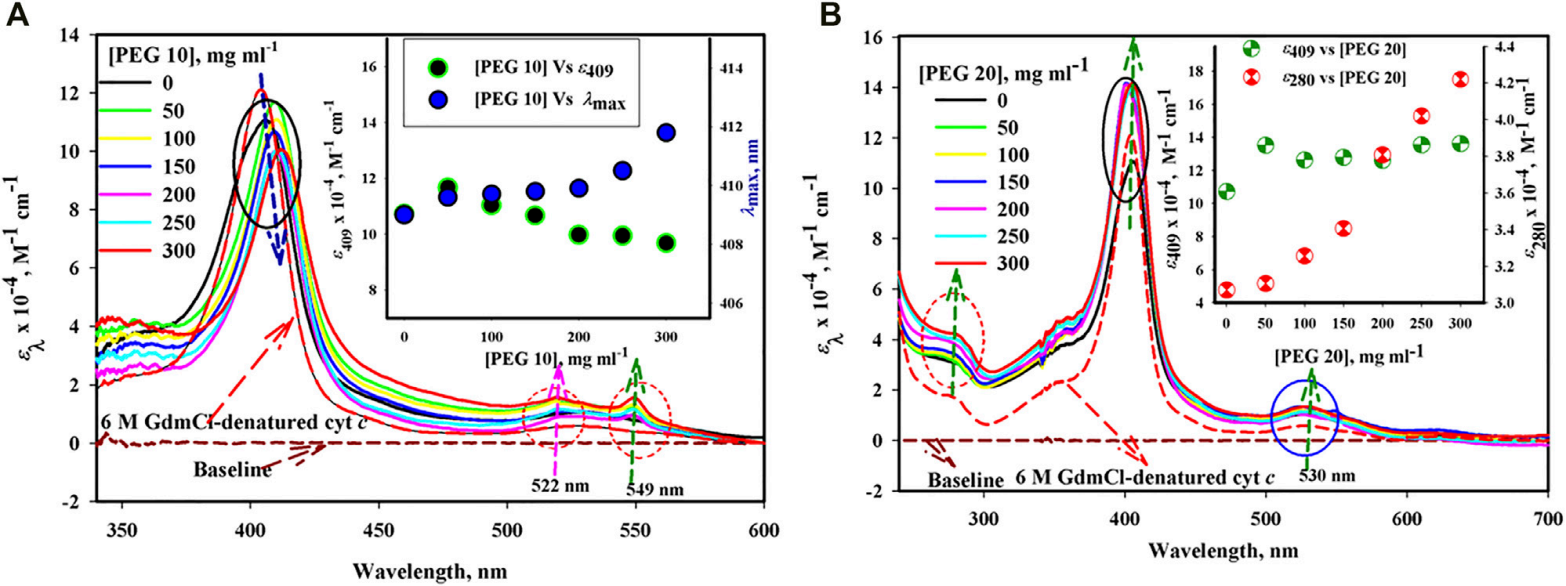
Absorption spectra of cyt *c* in the presence of various concentrations of **(A)** PEG 10 and **(B)** PEG 20. Inset of Figure **(A)** gives plot of *ε*_409_ and *λ*_max_ against [PEG 10]. Inset of Figure **(B)** gives plot of *ε*_409_ and *ε*_280_ against [PEG 20].

The UV–visible absorbance spectra of cyt *c* in the absence and presence of different concentrations of PEGs 10 and 20 are shown in Figures 1A,B. Figure 1A shows significant effect on the Soret region (decrease in the absorption coefficients at 409 nm (*ε*_409_) with red shift) and results in two peaks in oxy-deoxy regions (522 and 549 nm) of the protein in the presence of PEG 10, which was maximum at high concentrations. The inset of the figure depicts plots of the *ε*_409_ and *λ*_max_ (wavelength at which absorbance is maximum) versus [PEG 10], the concentration of PEG 10 in mg ml^−1^.

On the contrary, the protein in the presence of PEG 20 under similar conditions shows an increase in *ε*_409_ without any shift in the wavelengths and the single oxy-deoxy band around 530 (see Figure 1B). Also, the protein in the presence of PEG 20 (on increasing concentration) shows an increase in the absorption band around 280 without any shift in wavelengths. The figure also shows a decrease in the absorption coefficient of the protein (cyt *c*) around 280 and 530 nm and an increase in the absorbance around 409 nm with blue shift in the presence of 6 M GdmCl. The inset of Figure 1B shows a plot of *ε*_410_ versus [PEG 20].

### 3.2 Circular Dichroism Measurements

#### 3.2.1 Far-UV CD

**FIGURE 2:**
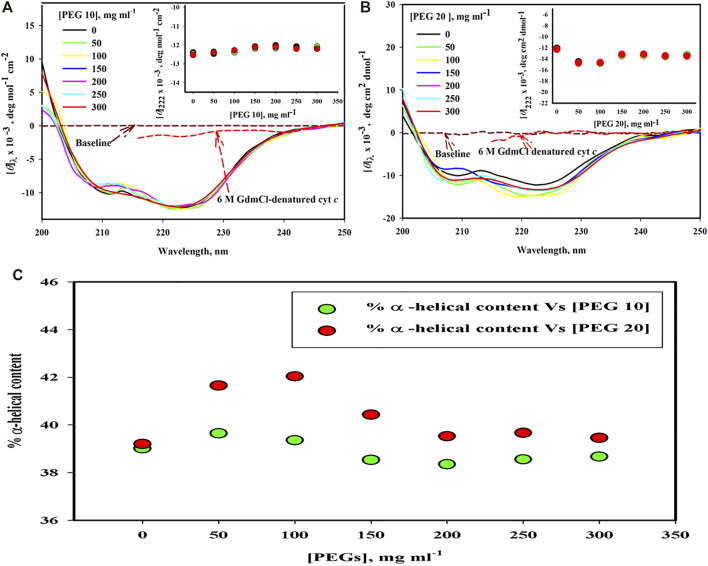
Far-UV CD spectra of cyt *c* in the presence of various concentrations of **(A)** PEG 10 and **(B)** PEG 20. Inset of Figure **(A)** gives plot of [*θ*]_222_ against [PEG 10]. Inset of Figure **(B)** gives plot [*θ*]_222_ against [PEG 20]. **(C)** α-helical content measured from [*θ*]_222_ values of far-UV CD spectra of the cyt *c* in the absence and presence of various concentrations of PEGs 10 and 20.

Figure 2A,B shows far-UV CD spectra of the protein treated with PEG 10 and PEG 20 at different concentrations (0–300 mg ml^−1^). The spectra of the denatured cyt *c* treated with 6 M GdmCl is also shown in this figure. Plot of [*θ*]_222_ versus [PEG 10] and [*θ*]_222_ versus [PEG 20] are shown in the insets of (A and B), where the red, green, and black circles signify data points of triplicate measurements (for data accuracy in measuring α-helical content). Figure 2C depicts the percentage change in the α-helical content of the protein as a function of the concentration of PEGs (PEGs 10 and 20). The percentage of α-helical content calculated from the values of [*θ*]_222_ using the equation from the study by Morrisett et al. (1973) is given in Table 1.

**TABLE 1:** Percentage of α-helical content of cyt *c* in the absence and presence of different concentrations of PEG 10 and PEG 20, estimated from the far-UV CD measurements at 222 nm^a^.

Crowder concentrations (mg ml^−1^)	PEG 10–induced change in % α-helix of cyt *c* [*θ*]_222_ ^b^	PEG 20–induced change in % α-helix of cyt *c* [*θ*]_222_
0	39.5 (±1.5)	39.8 (±1.5)
50	39.6 (±1.5)	41.65 (±1.6)
100	39.35 (±1.6)	42.04 (±1.3)
150	38.53 (±1.5)	40.43 (±2.0)
200	38.35 (±1.4)	39.52 (±1.0)
250	38.55 (±1.5)	39.7 (±1.65)
300	38.67 (±1.5)	39.45 (±1.43)

a Values of α-helical content were estimated using equations of Morrisett et al. (1973).

b Values in the parenthesis represents mean error from multiple measurements.

#### 3.2.2 Soret-CD

**FIGURE 3:**
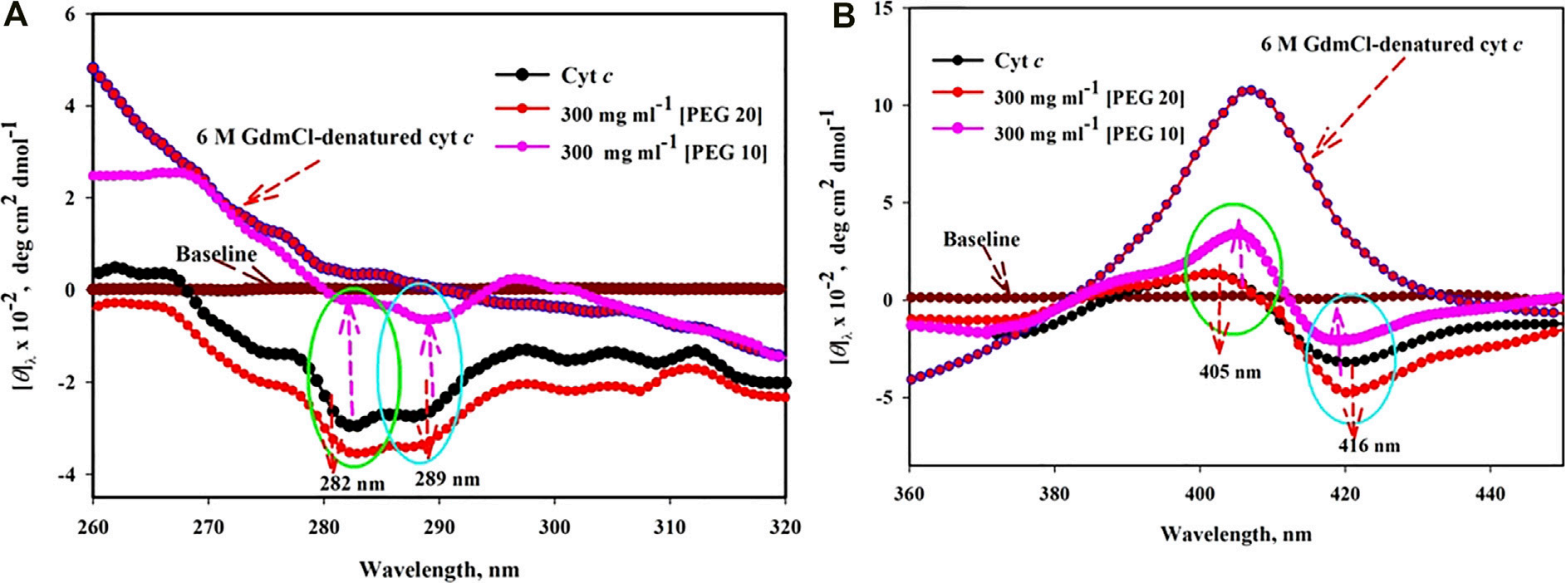
Near-UV CD **(A)** and Soret CD **(B)** spectra of cyt *c* in the presence of highest concentration (300 mg ml^−1^) of PEG 10 and PEG 20.

The UV–vis absorption results (Figure 1A,B) were further confirmed by Soret-CD spectra of cyt *c* in the absence and presence of PEGs 10 and 20 (see Figure 3A). Soret CD spectra of cyt *c* in the presence of 300 mg ml^-^1 of PEGs 10 and 20 and 6 M GdmCl are depicted in Figure 3A. This figure illustrates that the protein treated with 300 mg ml^−1^ of PEG 10 has a remarkable increase in the values of [*θ*]_405_ and decrease in the values of [*θ*]_416_. On the other hand, the protein treated with 300 mg ml^−1^ of PEG 20 results in insignificant change at [*θ*]_405_ and an increase in the values of [*θ*]_416_. 6 M GdmCl has significant effect over the protein which shows an increase in the value [*θ*]_405_ and decrease in the values of [*θ*]_416_, resulting from complete denaturation of the protein.

#### 3.2.3 Near-UV CD

Figure 3B shows near-UV CD spectra of cyt *c* in buffer (shows two negative peaks at 282 and 289 nm), in the presence of 300 mg ml^−1^ of PEG 10 and PEG 20 and 6 M GdmCl. The decrease and increase in the CD signals of the protein around [*θ*]_282_ and [*θ*]_289_ can be observed due to PEG 10 and PEG 20, respectively. Moreover, the CD signals of the protein around [*θ*]_282_ and [*θ*]_289_ are completely lost in the presence of 6 M GdmCl.

### 3.3 Size Distribution Measurements by Dynamic Light Scattering

**FIGURE 4:**
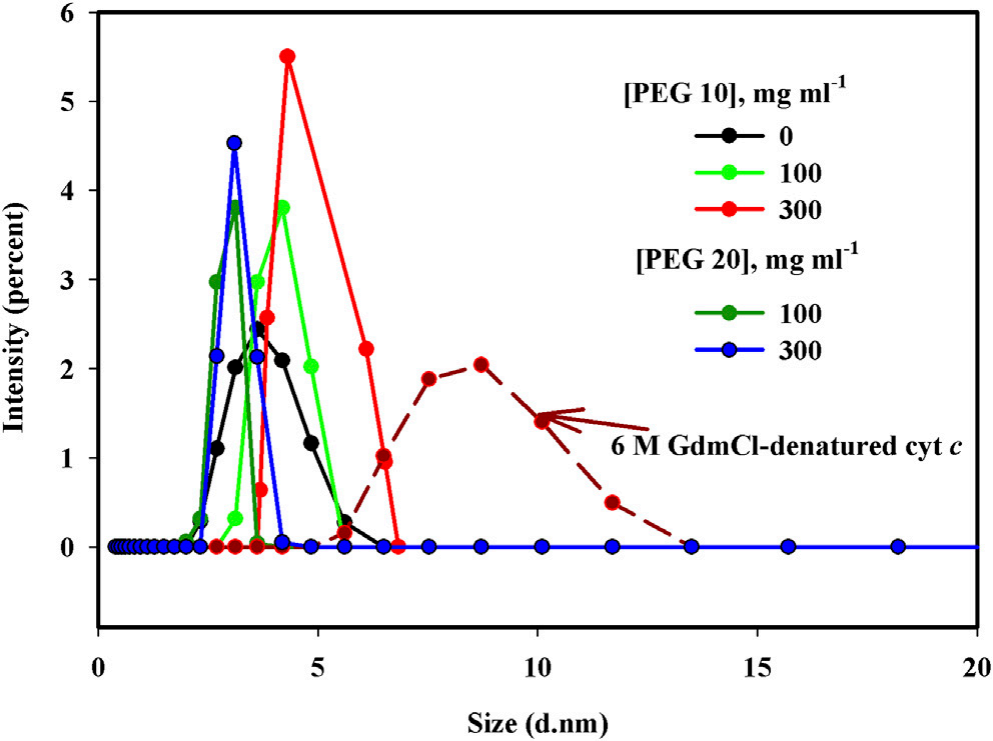
Particle size measurements (d.nm) of cyt *c* in the presence of 0, 100, and 300 mg ml^−1^ of PEG 10, PEG 20, and 6 M GdmCl.

Figure 4 shows the size distribution measurements of cyt *c* in the presence of buffer, crowders (PEG 10 and 20), and 6 M GdmCl. Hydrodynamic radius (*R*_h_) values in angstroms of the protein under different solvent conditions (buffer, crowders, and denaturant) are presented in Table 2.

**TABLE 2:** *R*_h_ and *V*_h_ values of cyt *c* in the presence of different concentrations—lowest and highest concentrations of PEG 10 and PEG 20^a^.

Crowder concentrations (mg ml^−1^)	PEG 10–induced change in *R*_h_, Å	PEG 20–induced change in *R*_h_, Å
**0**	16.5 (±0.8)	16.5 (±0.8)
**100**	18.4 (±1.2)	15.4 (±0.86)
**300**	20.8 (±1.5)	14.8 (±0.95)

a Values in the parenthesis represent mean error from multiple measurements.

### 3.4 Binding Measurements

#### 3.4.1 Isothermal Titration Calorimetric Measurements

**FIGURE 5:**
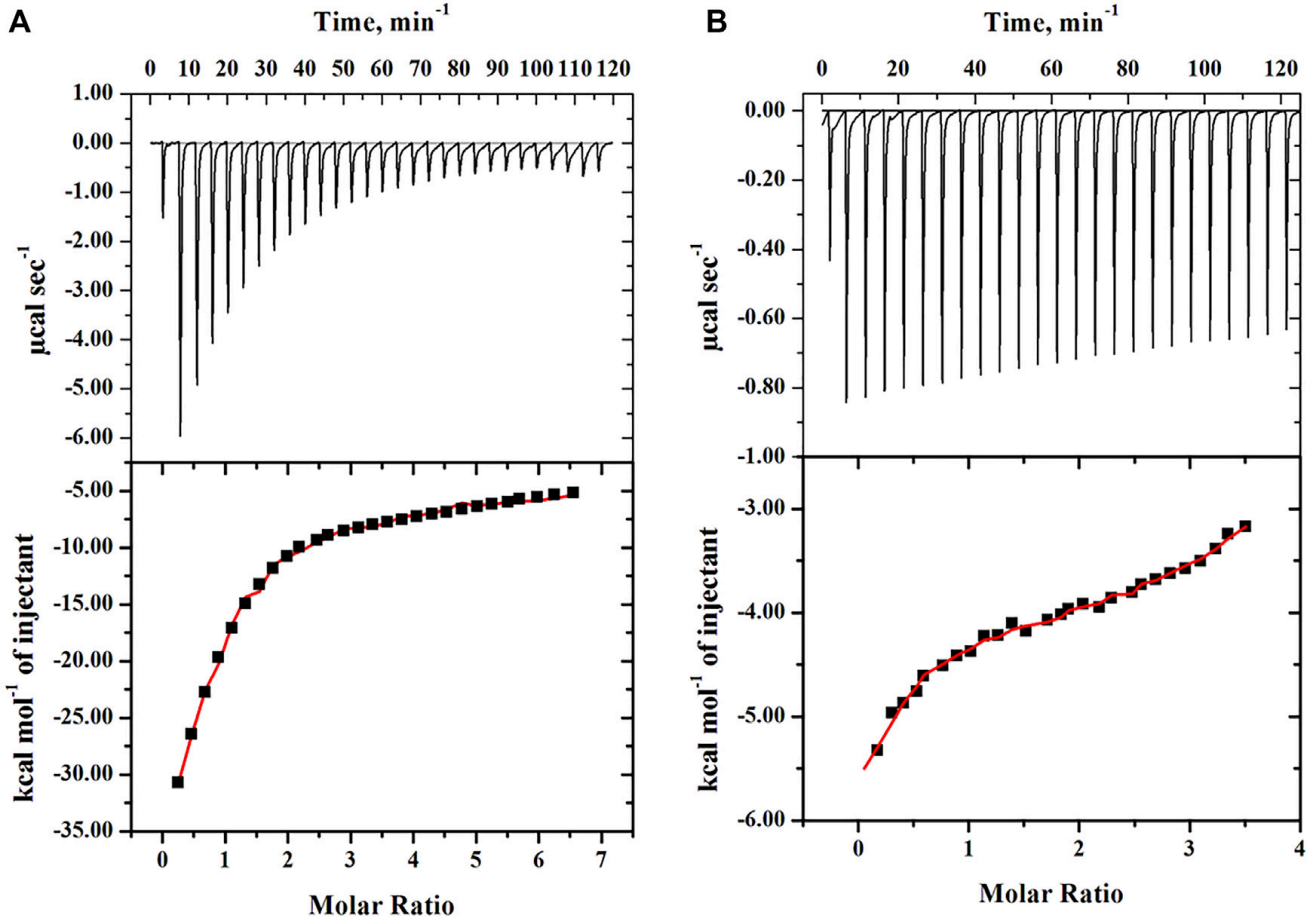
Calorimetric binding measurements of cyt *c* titrated by **(A)** PEG 10 and **(B)** PEG 20 at 25°C. The upper panel shows the observed heat after each injection. For each figure, the lower panel represents the integration of the data shown in the upper panel plotted as a function of the molar ratio of the protein to the PEG.

To measure the thermodynamic binding parameters and to know and understand the type of interaction and their strength between PEGs (10 and 20) with the protein (cyt *c*) in similar settings, calorimetric measurements were carried out. For the interaction studies, the crowders were loaded in the syringe (separate experiment was done for both) followed by titrations into the cell with cyt *c*. The top section of Figures 5A,B provides the raw data, where the power is plotted against the time. The lower panel in these figures displayed the normalized power of the raw data with respect to the amount of injection (kcal mol^−1^) against the molar ratio of crowding agents injected. Values of thermodynamic parameters, the binding association constant (*K*_a_), equilibrium dissociation constant (*K*_d_), standard enthalpy (Δ*H*˚), and standard entropy (Δ*S*˚) of both crowder-cyt *c* systems are presented in Table 3. This table also shows standard Gibbs free energy changes (Δ*G*˚) of the bi-molecular reactions calculated using Eq. 2.

**TABLE 3:** Calorimetric binding parameters estimated from ITC measurements on interaction of PEGs (PEG 10 and PEG 20 kDa) with cyt *c* at 298 K (25°C)^a^.

Thermodynamic parameters (units)	*K*_a_ (M^−1^)	∆*H* ^o^ (cal mol^−1^)	∆*S* ^o^ (cal mol-1deg-1)	∆*G* ^o^ (cal mol^−1^)	*K*_*d*_ (M)
	PEG 10-cyt *c*		
Step 1	17.6 × 10^4^ **(**± 0.91 × 10^4^)	−4,402 (±91.5)	9.24	−7.2 × 103 (±0.092 × 103)	0.57 × 10^−5^
Step 2	6.39 × 10^4^ **(**± 0.35 × 10^4^)	639.8 (±151)	24.1	−6.52 × 103 (±0.15 × 103)	0.16 × 10^−4^
Step 3	6.09 × 10^3^ **(**± 0.27 × 10^3^)	−12.17 × 103 (±372)	23.5	−19.2 × 103 (±0.37 × 103)	0.16 × 10^−3^
**PEG 20-cyt *c***					
Step 1	10.01 × 10^4^ **(**± 0.1 × 10^4^)	−7.404 × 103 (±0.1 × 103)	−1.93	−6.829 × 103 (±0.1 × 103)	0.99 × 10–5
Step 2	9.93 × 10^4^ **(**± 0.08 × 10^4^)	−3.57 × 103 (±0.5 × 103)	10.9	−6.9 × 103 (±0.5 × 103)	0.10 × 10–4
Step 3	10.01 × 10^4^ **(**± 0.08 × 10^4^)	−2.196 × 103 (±1.02 × 103)	15.5	−6.82 × 103 (±1.02 × 103)	0.99 × 10–5
Step 4	10.2 × 10^4^ **(**± 0.08 × 10^4^)	−7.15 × 103 (±0.8 × 103)	−1.06	−6.83 × 103 (±0.8 × 103)	0.98 × 10–5

a A ± signifies the mean error of each parameter measured from triplicate measurements.

#### 3.4.2 Molecular Docking Studies

**FIGURE 6:**
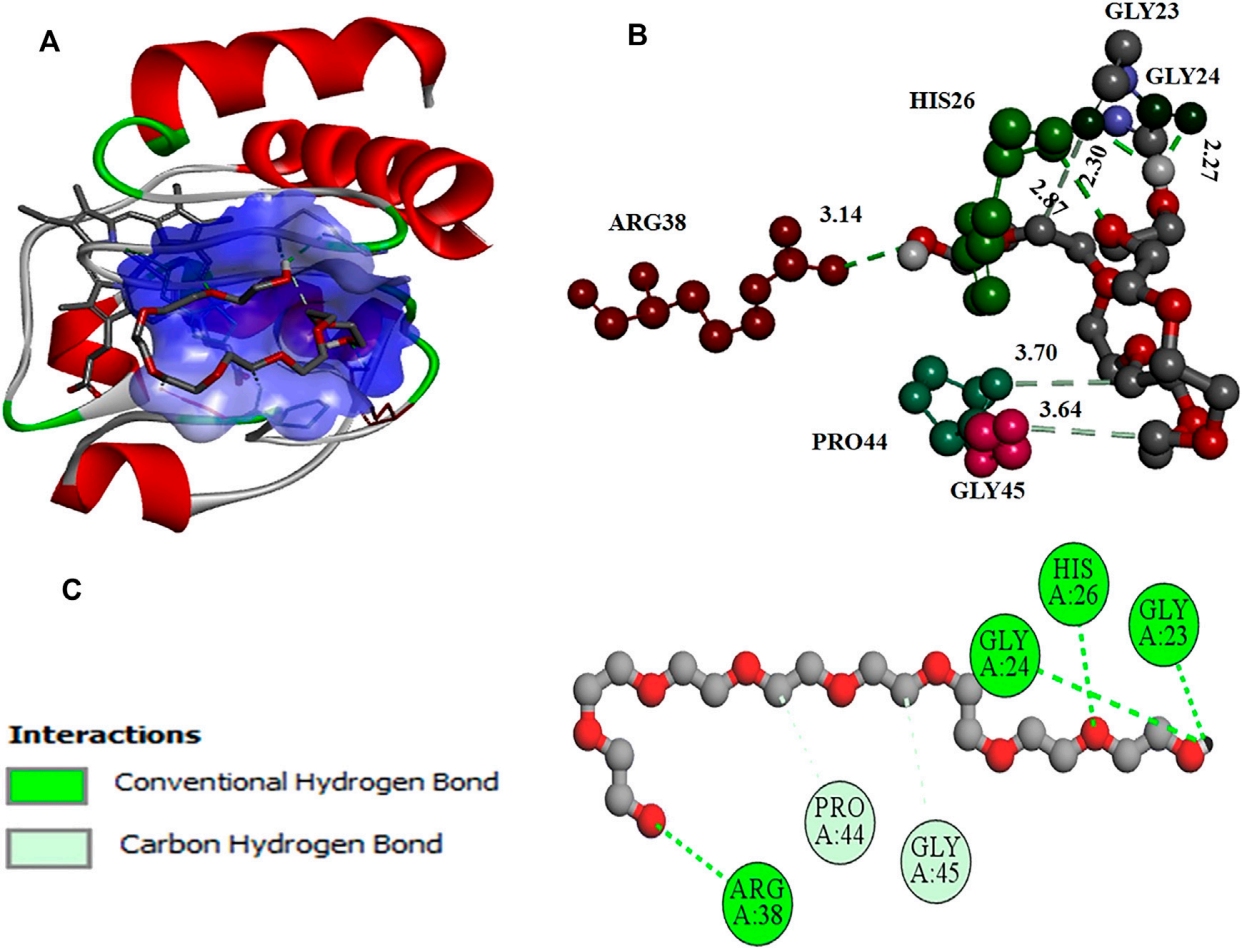
**(A)** Surface area and binding site on the cyt *c* (cartoon model, red) for PEG 10 (stick model, gray). **(B)** Various amino acid residues (stick model) of the protein interacting with PEG 10 (ball and stick model, gray) with given bond lengths. **(C)** 2D representation of various types of interactions of amino acid residues with PEG 10.

**FIGURE 7:**
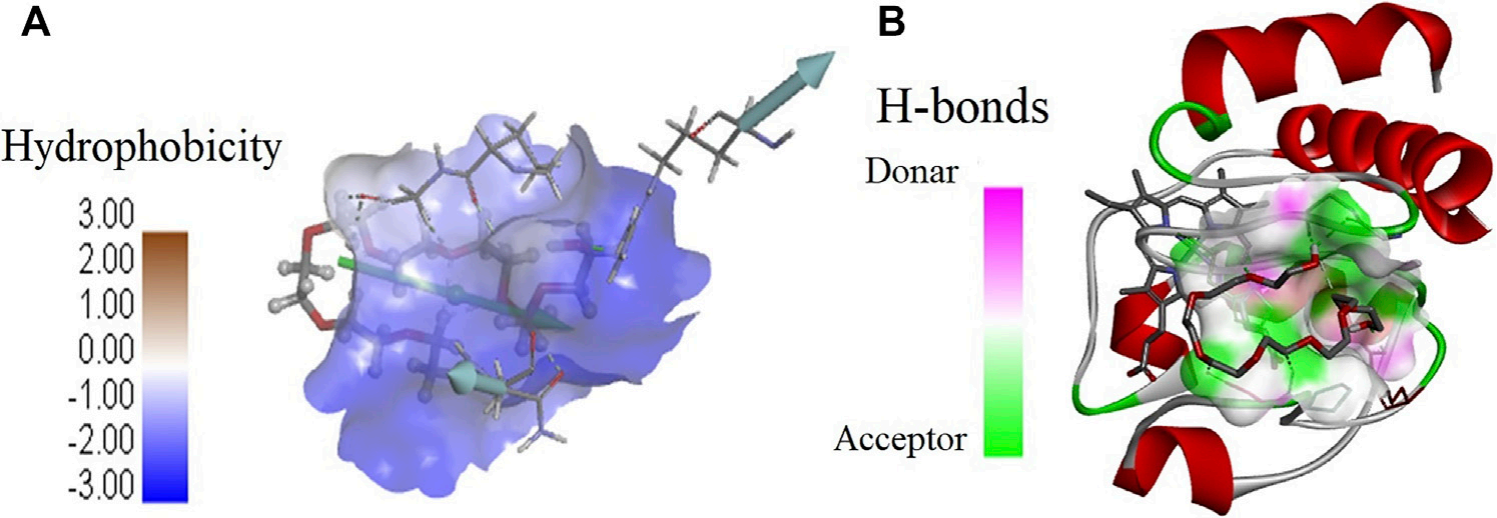
Values of **(A)** hydrophobicity and **(B)** H-bonds (acceptor and donor) on the surface of cyt *c* interacting with PEG 10. **(A)** Porcupine plot shows hydrophobic moment outlines in the form of an arrow directing possible amphiphilic α-helical regions on the protein on interaction of PEG 10.

Further molecular docking studies were made to know the binding site of crowders (PEGs 10 and 20) on cyt *c* (PDB ID: 1hrc), the residues of protein interacting, and the types of interactions occuring. Figure 6 shows interaction of PEG 10 with cyt *c*, where **(A)** shows the binding site and **(B** and **C)** shows the residues of the protein interacting with the crowder and types of interactions occurring. Figure 7A depicts the surface area of the protein based on hydrophobicity, that is, solvent-accessible surface area assists in finding complementary pose of binding between two interacting molecules and the arrow gives the porcupine plot which shows the region influenced by the ligand. Figure 7B represents the hydrogen (H)- bonds between the donor (protein residues) and acceptor residues of the protein interacting with the ligand (PEG 10).

**FIGURE 8:**
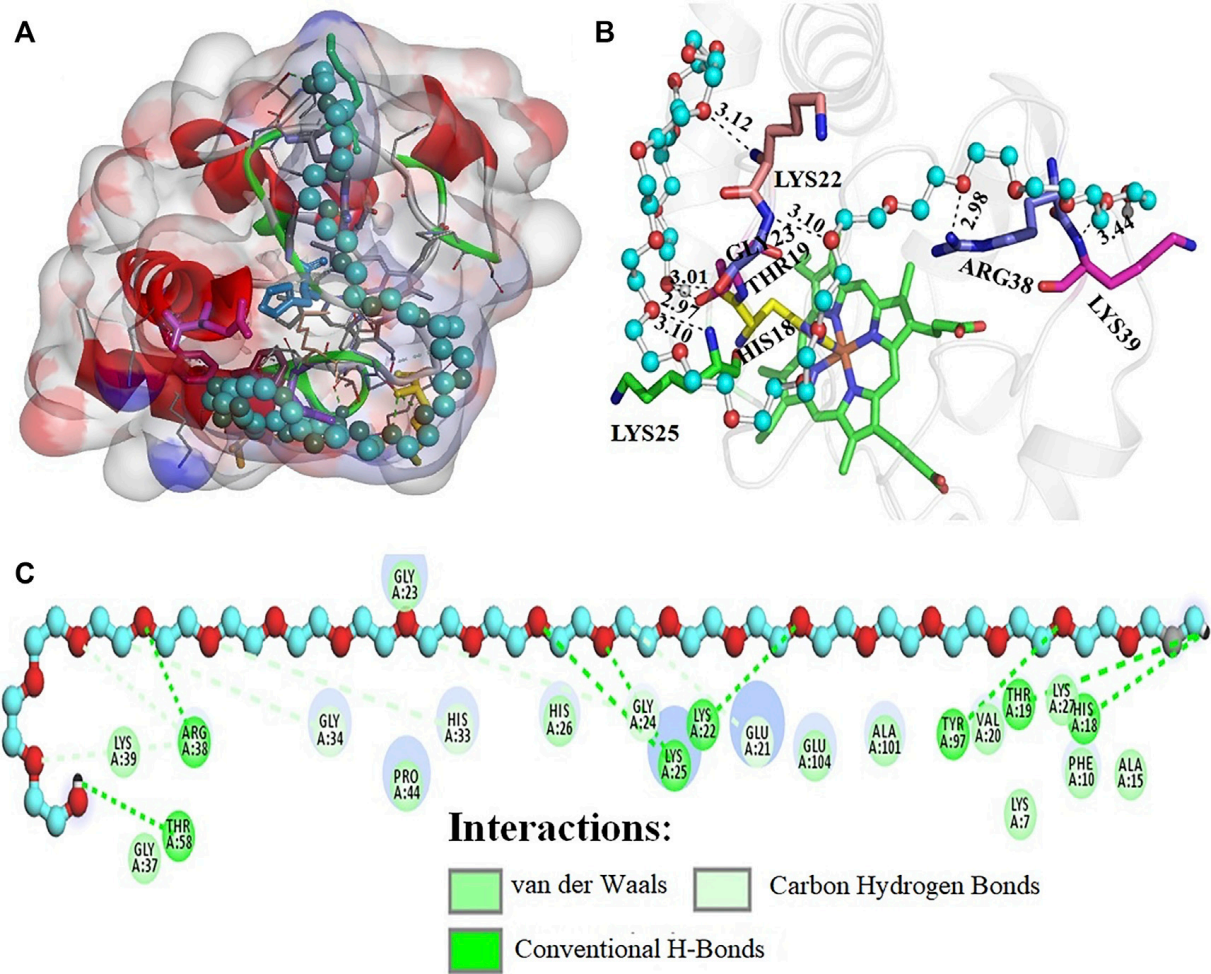
**(A)** Surface area and binding site on the cyt *c* (cartoon model, red) for PEG 20 (ball and stick model, blue). **(B)** Various amino acid residues (stick model) of the protein interacting with PEG 20 (ball and stick model, blue) with given bond lengths. **(C)** 2D representation of various types of interactions of amino acid residues with PEG 20.

**FIGURE 9:**
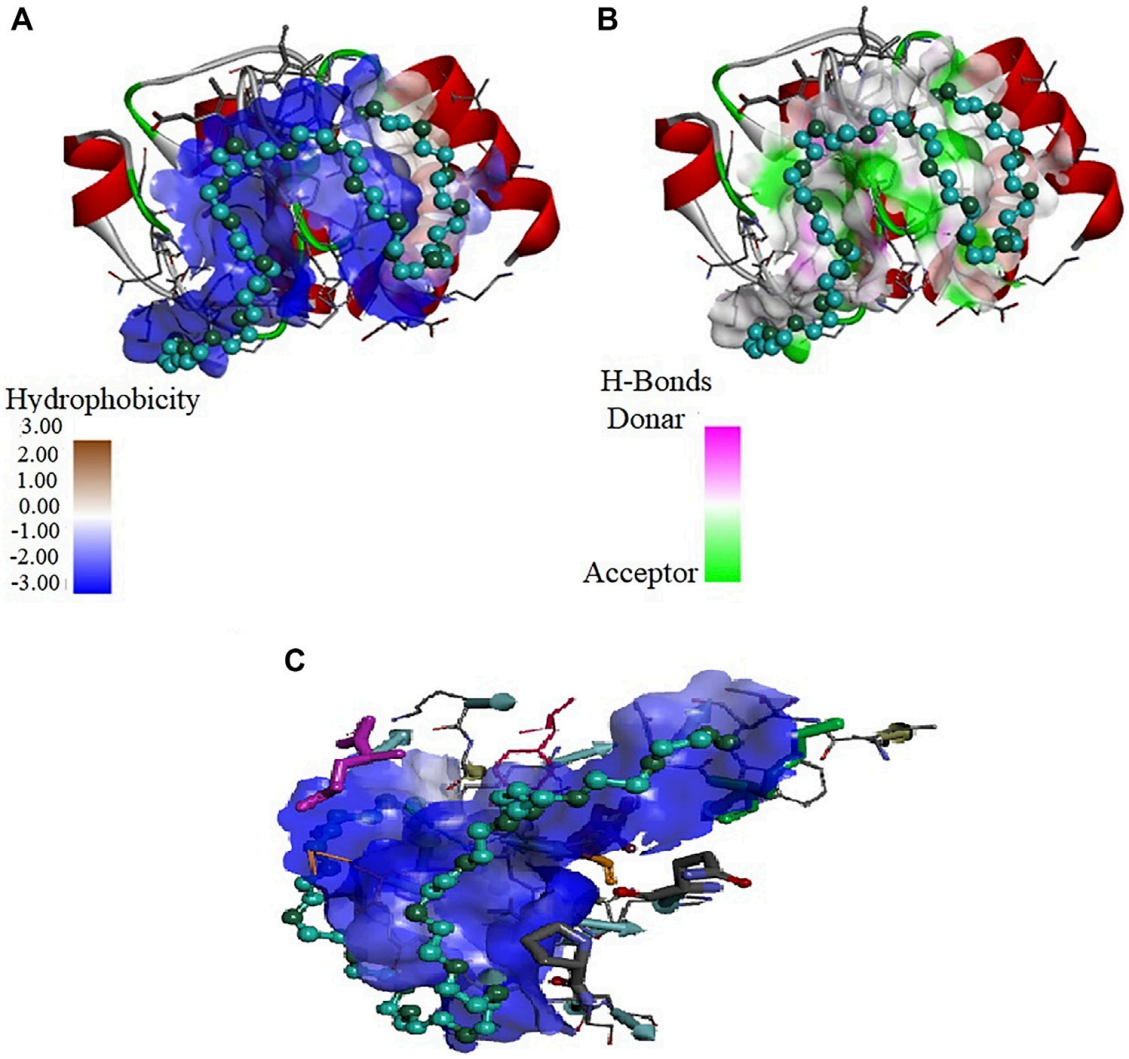
Values of **(A)** hydrophobicity and **(B)** H-bonds (acceptor and donor) on the surface of cyt *c* interacting with PEG 20. **(C)** Porcupine plot shows hydrophobic moment outlines in the form of an arrow directing possible amphiphilic α-helical regions on the protein on interaction of PEG 20.

Figure 8 depicts the interaction of PEG 20 with cyt *c*, where **(A)** shows the binding site and **(B and C)** shows the interacting residues of the protein with PEG 10 and types of interactions occurring. Figure 9A depicts the surface area of the protein based on hydrophobicity, that is, solvent-accessible surface area assists in finding complementary pose of binding between two interacting molecules (PEG 20-cyt *c*). Figure 9B represents the hydrogen bonds between donor and acceptor residues on the protein interacting with the ligand (PEG 20). Figure 9C is a porcupine plot of protein with crowder (PEG 20).

## 4 Discussion

Our research group has exploited a spectrum of different sizes of PEG, other crowding agents (ficoll and dextran), and some mixture solutions of crowders to measure their effect on heme-proteins in order to infer the significance of macromolecular crowders of different shapes and sizes in the cellular environments (Parray et al., 2017; Nasreen et al., 2018; Parray et al., 2019; Parray et al., 2020a; Parray et al., 2020b; Parray et al., 2020c; Parray et al., 2020d; Nasreen et al., 2020; Parray et al., 2021a; Parray et al., 2021b). The goal of the study was always to concentrate mostly on the advances through *in vitro* and *in silico* methods to measure the implications of varying sizes of macromolecular crowders on biophysical characteristics and conformational changes in proteins. Inert polymer solutions may imitate the state of crowding *in vitro* to understand *in vivo* or cellular conditions, according to information gathered from protein–molecule interactions. Proteins are subjected to different macromolecules inside living cells and perform their function even after interaction with molecules that vary in size, shape, and concentration. In this study, we used two different broad-range–sized PEGs, in which one is the double of the other, including PEGs 10 and 20 kDa.

UV–visible spectroscopy is a steady essential method of spectroscopy to investigate the conformational changes of proteins (Nienhaus and Nienhaus, 2005). The native ferri-cyt *c* shows a peak around 410 nm in the Soret region (Dixon et al., 1931), which is because of the presence of porphyrin (a chromophore), a perceptive probe for observing any change in the heme environment (Schejter et al., 1996; Fedurco et al., 2004). The size-dependent effect of PEGs on the structure of cyt *c* can be seen in the broad absorption spectra of Figure 1A,B. Cyt *c* shows different absorption bands, including around 280 nm (due to the presence of phenyl groups of Tyr and Trp residues), Soret region around 405–410 nm (arises because of heme-globin interaction and iron state), and an oxy-deoxy tiny band(s) in the region of wavelength 500–600 nm (Myer and Kumar, 1989). The sharp Soret band around 410 nm is the signature of the native cyt *c* conformation (Dixon et al., 1931). The Soret band position and peak shape are completely dependent on the geometric position of the iron atom present in the prosthetic group (Boffi et al., 1994; Mahato et al., 2010). As can be seen in the inset of Figure 1A, an increase in Soret absorption without any change in *λ*_max_ occurs at low concentrations of PEG 10 (50 mg ml^−1^), indicating a stronger heme–protein interaction, while this crowder at higher concentrations (100, 150, 200, 250, and 300 mg ml^−1^) causes a decrease in the absorption with red shift in the spectrum suggesting that a major change in the protein–heme interaction occurred (Fedurco et al., 2004; Tomášková et al., 2010; Mondal and Das, 2018). Formation of two bands around 525 (α-peak) and 549 (β-peak) and increase in absorbance caused by the crowder in the oxy-deoxy region confirm that the iron state in the heme group is changed to reduced form when exposed to PEG 10, quite the opposite of the native state (the protein in buffer) which shows only one peak around 530 nm (Fedurco et al., 2004). On the contrary, the Soret band of cyt *c* in the presence of PEG 20 (50 mg ml^−1^) showed a slight increase in the absorbance without any red shift; however, adding more amount of PEG 20 (100, 150, 200, 250, and 300 mg ml^−1^) leads to a slight increase in the intensity (can be seen in the inset of Figure 1B) but without any shift in the wavelength. The oxy-deoxy band shows a slight increase in the absorbance around 530 nm and without showing change in the number of bands of cyt *c* in the presence of PEG 20, which confirms the iron state in the heme group is in the oxygenated state as that of the native protein (in buffer). Moreover, Trp and Tyr environment changes toward more non-polar environment (i.e., buried more than the native protein), as can be seen in the inset of Figure 1B, where *ε*_280_ of the protein increases on increasing the concentration of PEG 20. The absorption spectrum of cyt *c* is perturbed by 6 M GdmCl (see Figure 1), which has opposite effects to that of PEG 20. The results suggested that PEG 20 assists in the formation of the tertiary structure and heme-globular interactions of the protein, in contrast to PEG 10 and 6 M GdmCl.

To validate conclusions drawn from the near-UV absorption measurements (see Figures 1A,B), additional probes including 1) Soret CD: to monitor the changes in the intramolecular interactions such as heme–protein, heme–Phe, and Met–Fe (Figure 3A) and 2) near-UV CD: to monitor changes in aromatic amino acid (Tyr and Trp) environments of cyt *c* in the presence of PEG 10 and 20 (see Figure 3B) were used.

The probe [*θ*]_405_ is used to measure changes in the heme–globin interaction and [*θ*]_416_ is used to monitor changes in the interaction strength of Met 80-Fe and Phe82 with heme (Harbury et al., 1965; Pielak et al., 1986; Santucci and Ascoli, 1997; Kagan et al., 2005; Zaidi et al., 2014). When protein was exposed to 300 mg ml^−1^ of PEG 10, there was a considerable increase in the CD signal (positive peak) of cyt *c* at [*θ*]_405_; however, no change has been observed in the probe when the protein was exposed to 300 mg ml^−1^ of PEG 20 (see Figure 3B). Changes observed at [*θ*]_416_ show a small shift toward lower wavelength with significant drop in the MRE of the protein exposed to 300 mg ml^−1^ of PEG 10; in contrast, there was an increase in the ellipticity with small shift in wavelengths when protein was exposed to 300 mg ml^−1^ of PEG 20. The figure showed that the Soret CD spectra of the protein in the presence of 6 M GdmCl completely disappeared around 416 nm, and CD signals around 405 nm were increased to maximum. Such structural changes in the protein suggest that the protein conformation is disrupted (Santucci and Ascoli, 1997), causing changes in the heme–protein, Met–Fe, and heme–Phe interactions in the presence of 6 M GdmCl and PEG 10; however, PEG 20 stabilizes such intra-molecular interactions in the protein (Parray et al., 2020c; Parray et al., 2021a). Near-UV CD probe was used to monitor the changes in the tertiary structure and to discern the effects of PEGs (10 and 20) on the local environment of Tyr and Trp (aromatic amino acids), (Kelly and Price, 2000). It can be seen in Figure 3A that the spectrum of the native cyt *c* in the near-UV CD shows two negative peaks at 282 and 289 nm, an observation in agreement with that reported earlier (Moosavi-Movahedi et al., 2003; Moza et al., 2003). These two negative CD signatures attributes to a stiff packed structure (tertiary) in the environments of Tyr(s) and Trp59 which is in contact with the heme group of the protein *via* one of its propionate groups (Davies et al., 1993). The results showed that PEG 10 decreases the CD signals and affect the signatures ([*θ*]_282_ and [*θ*]_289_) significantly, though PEG 20 has opposing effects, that is, leads to an increase in the CD signals and signature values. The above observations from the results suggest that higher crowder concentration of PEG 10 perturbs the tertiary structure in terms of the native environment of tyrosine and tryptophan; in contrast, PEG 20 leads to more protein compaction (aromatic residues move toward the non-polar environment (Antosiewicz and Shugar, 2016). The spectrum of the 6 M GdmCl-denatured-cyt *c* shown in Figure 3A is used as a reference for the unfolded protein. It is seen in this figure that GdmCl causes complete disappearance of [*θ*]_282_ and [*θ*]_289_ signatures.

The far-UV CD technique was used to examine changes in secondary structural elements and for determining the protein secondary structure (Kelly and Price, 2000). However, the estimation by the technique is not accurate enough; one should be cautious and do experiments in triplicate measurements to stablize the accuracy. In cyt *c*, there are two strong CD signals that characterize the properties of all α-proteins (Greenfield and Fasman, 1969). This technique was used to monitor changes in the secondary structure of cyt *c* in the presence of different concentrations of different sizes of PEG (PEGs 10 and 20) and in the presence 6 M GdmCl (Figure 2A,B). The signatures at 222 and 208 nm in the far-UV CD spectrum of proteins are the characteristics of all α-proteins. These signatures can be seen in the far-UV CD spectrum of cyt *c* (Figures 2A,B) (Moosavi-Movahedi et al., 2003; Moza et al., 2006; Khan et al., 2016). From the results, it was observed that PEG 10 has no significant effect on the secondary structure of cyt *c* at all concentrations (Figures 3A,C). On the other hand, PEG 20 increases the secondary structure (α-helical content) of the protein at lower concentrations of the crowder (Figure 2C; Table 1); however, insignificant effect was observed at its higher concentrations (Figure 2C; Table 1). The percentage of secondary structural content is provided in Table 1 which confirms that the secondary structure was not perturbed both in the presence of PEG 10 and PEG 20; however, PEG 20 stabilizes the structure of the protein. Percentage of α-helix was estimated from the values [*θ*]_222_, using the equation from the study by Morrisett et al. (Morrisett et al., 1973). The observed α-helical content is in agreement with those reported earlier (Qureshi et al., 2003; Khan et al., 2011; Parray et al., 2020c; Parray et al., 2021a).

To know the diffusion coefficient, dimmer formations, and change in size of macromolecules in various solvent systems, DLS is an approach to be used (Stetefeld et al., 2016). Size distribution measurements of cyt *c* in the presence of buffer, PEG 10, PEG 20, and 6 M GdmCl were made (see Figure 4). The software Zetasizer Ver. 7.13 of Malvern Panalytical was used to analyze the values of hydrodynamic size from the plots shown in Figure 4 where the diameter (nm) of each sample is plotted against Intensity. The obtained results in diameter were converted into hydrodynamic radius (*R*_h_), given in Table 2. It can be concluded that *R*_h_ values of the wild-type cyt *c* (in the buffer) was 1.65 nm (16.5 Å) and in 6 M GdmCl was equal to 3.85 nm (38.5 Å), which are nearly equivalent to the values reported previously (Khan et al., 2011; Parray et al., 2020a; Parray et al., 2021a). The *R*_h_ of cyt *c* increases from 1.65 nm (16.5 Å) in the absence of crowder to 1.84 nm (18.4 Å) and 2.08 nm (20.8Å) in the presence of 100 and 300 mg ml^−1^ of PEG 10, respectively. Values of *R*_h_ of the protein in the presence of 100 and 300 mg ml^−1^ are 1.54 nm (15.54 Å) and 1.48 nm (14.8 Å), respectively. These observations suggest that PEG 10 denatures cyt *c* (increases the hydrodynamic size) and PEG 20 results in structure compaction at high concentrations (decreases the hydrodynamic size). Thus, it can be said that PEG 10 results in the perturbation of the tertiary structure of cyt *c* without change in the secondary structure; however, PEG 20 leads the protein to a more structured form (Qu et al., 2012; Parray et al., 2020c; Das and Sen, 2020; Parray et al., 2021b).

To know whether there is any specific interaction between protein and crowders, we performed ITC measurements and molecular docking studies of the protein with the crowders (PEGs 10 and 20). ITC measurements showed that both crowders (PEGs 10 and 20) bind with cyt *c* (see Figure 5A,B). ITC data were analyzed using different binding models. Analysis showed that the best fit of the data is obtained when they were fitted according to the three-step sequential binding site model for the PEG 10-cyt *c* system and the four-step sequential binding model for the PEG 20-cyt *c* system. This analysis of data gave thermodynamic parameters (see Table 3). For one-site and two-site binding models, the value of stoichiometry (n) can be achieved directly from the plot of heat change per mole of the ligand versus the molar ratio of ligand to protein (see lower panels in Figure 5). The stoichiometry (n) of the bi-molecular interaction, which is the equivalence point of the molar ratio, determined directly from the figures, is approximately 2.5 for the PEG 10-cyt *c* system (Figure 5A), which is greater than that (about 1.2) of the PEG 20-cyt *c* system (Figure 5B). However, in sequential binding, where ligand molecules bind to the receptor at independent sites (more than 2 molecules bind at multiple sites) or identical sites (more than 2 molecules bind at almost the same spot), it is hard to determine sites at which ligand molecules bind with the receptor (Freyer and Lewis, 2008). There are various studies of protein–ligand binding that showed that ITC thermograms were best described using the sequential binding site model (Popovych et al., 2006; Rajarathnam and Rosgen, 2014; Krainer and Keller, 2015; Yu et al., 2017; Parray et al., 2021a).

Calorimetry is the best method to get the thermodynamic parameters directly when a macromolecule shows interaction with ligand, and in this bi-molecular reaction change in enthalpy (Δ*H* ^°^) as a probe to define the degree of an interaction (Ladbury and Chowdhry, 1996), the change in entropy (Δ*S* ^°^) on complex formation defines the global thermodynamic feature of a system; greater Δ*S* ^°^ signifies that the degree of freedom of the system is increased and a decrease in the values of Δ*S* ^°^ signifies that the degree of freedom of the system is decreased (Du et al., 2016). The change in free energy (Δ*G* ^°^) defines the spontaneity of the reaction and binding affinity (*K*_a_) measures the strength of the intermolecular interactions; intermolecular soft interactions are non-covalent in nature, which include hydrogen bonding, electrostatic interactions, van der Waals forces, and hydrophobic interaction (Ladbury and Chowdhry, 1996).

Table 3 gives values of the thermodynamic binding parameters for cyt *c*-PEG 10 and cyt *c*-PEG 20 systems. It can be seen in this table that the observed value of Δ*G*˚ is highly negative in the case of PEG 10-cyt *c* and less negative in the case of PEG 20-cyt *c*, and *K*_a_ values are better in the former than the latter. The sum of the total Δ*G* ^o^ values for cyt *c*-PEG 10 and PEG 20-cyt *c* systems suggested that both the intermolecular interactions are spontaneous in nature. It can be seen in this table that the bi-molecular reaction of cyt *c*-PEG 10 is more exothermic in nature than the cyt *c*-PEG 20 reaction. The sum of the total Δ*S* ^o^ suggested that bi-molecular reaction (cyt *c*-PEG 10) gives maximum Δ*S*, which is double the sum of total Δ*S* ^o^ given by bi-molecular reaction (cyt *c*-PEG 20). Hence, the Δ*S* ^o^ values are greater in the system (cyt *c*-PEG 10) that shows an increase in the degree of freedom, consequently resulting in disordered (perturbed) protein (strong binding) (Du et al., 2016). In contrast, Δ*S* ^o^ values are smaller in the system (cyt *c*-PEG 20) that showed a decrease in the degree of freedom, resulting in ordered structure of the protein (weak binding).

*K*_a_ values given in the table suggest that PEG 10 showed greater binding than PEG 20 with cyt *c* in the first step (1 binding site). However, the sum of the total binding PEG 20 was greater because the surface area of PEG 20 is greater than that of PEG 10, and hence, it interacts with amino acids on the surface more than PEG 10 (see Figures 6, 8, and Table 4). *K*_d_ values given in Table 3 were calculated from *K*_a_; greater values of *K*_d_ signify weak binding. The results and analysis out of calorimetric binding studies show that PEG 10 strongly interacts with the protein (cyt *c*) compared to PEG 20; this conclusion was supported by molecular docking studies (see Figures 6, 8, and Table 4).

**TABLE 4:** Binding parameters obtained and various types of interactions observed in between crowders (PEG 10 and PEG 20) and cyt *c* by molecular docking.

Crowder molecule	Bonds	Interacting amino acid residues	Type of interaction	Bond distance (Å)	^b^ΔG* (kcal mol^−1^)	^c^ *K*_a_* (M^−1^)
PEG 10	1	Gly24	Conventional H-bonds	2.27	-4.1	9.93 × 10^2^
		^a^His26		2.87		
		^a^Gly23		2.30		
		^a^Arg38		3.14		
	1	^a^Pro44	Carbon hydrogen bond	3.70		
		Gly45		3.64		
PEG 20	1	Thr58	Conventional hydrogen bond	3.26	-3.2	2.2 × 10^2^
	1	^a^Arg38	Carbon hydrogen bond	2.95		
	1	Lys22		3.12		
	2	Lys25		3.10		
	1	Tyr97		2.97		
	1	Thr19		3.45		
	1	His18		3.10		
	1	^a^Gly23		3.97		
	1	Gly34		3.01		
	1	His33		4.12		
	1	^a^His26		4.54		
	2	Arg38		3.93		
	1	Glu 21		3.82, 3.87		
		Gly37	van der Waals interaction			
		^a^Pro44				
		Ala101				
		Glu104				
		Val20				
		Lys7				
		Phe10				
		Ala15				
		Lys27				
		Lys 39				
		Gly23				

a Interacts with both with PEG, 10, and PEG, 20.

b Binding energy.

c Binding affinity = 1/*K*_b_(1/binding constant).

The computational studies showed that PEG 10 gives a binding free energy (ΔG*) of –4.1 kcal mol^−1^ corresponding to a binding constant (*K*_a_*) of 9.93 × 10^2^, on interaction with Gly24, His26, Gly23, and Arg38 *via* conventional H-bonds and Pro44 and Gly45 *via* carbon-hydrogen bonds. In contrast, PEG 20 gives a binding free energy (ΔG*) of –3.2 kcal mol^−1^ corresponding to a binding constant (*K*_a_*) of 2.2 × 10^2^, on interaction with Thr58, Arg38, Lys22, Lys25 (2 bonds), Tyr97, Thr19, His18, and Gly23 *via* conventional H-bonds; Gly34, His33, His26, Arg38, and Glu21 *via* carbon-hydrogen bonds; and Gly37, Pro44, Ala101, and Glu104 (see Table 4) *via* van der Waals interactions. From the table, amino acids (His26, Gly23, Arg38, and Pro44) interact with both the crowder molecules (PEG 10 and PEG 20). Overall, the binding studies (both *in vitro* and *in silico*) confirm that PEG 10 strongly binds or interacts with cyt *c* in assessment of PEG 20, which has large size, stabilizing the protein. The strength of interaction and protein perturbation was observed to be crowder size dependent; large size of crowders showed either no perturbation or more stabilization, and small crowders show good binding and hence more destabilization.

Theoretical approaches for predicting the magnitude of crowding and confinement on macromolecular reactions make the assumption that these effects are primarily entropic in nature, that is, that they result from an increase in the configurational entropy of reactants (cyt *c*-PEG 10), maybe forming a transition state, and decrease in the configurational entropy of reactants (cyt *c*-PEG 20), due to crowding or confinement (Zhou et al., 2008). Other nonspecific interactions, such as electrostatic repulsion and attraction and hydrophobic attraction, have been identified from molecular docking results as likely to contribute significantly to total energetics in highly packed or confined systems.

Moreover, Figures 7, 9 provide values which measure solvent-accessible surface area based on hydrophobic nature of amino acids which assist in finding complementary pose of binding for the ligand molecules. These figures also depict the hydrogen bonds (donor and acceptor) formed by the residues of the protein during interaction with crowders (PEGs 10 and 20). Figures 7A, 9C showed porcupine plots which depict distinctive motions by high-magnitude arrows observed mostly in the flexible regions of the protein that are influenced by the interactions with ligands (Skopalík et al., 2008; Lertkiatmongkol et al., 2013). These also signify a hydrophobic moment profile which is applied to determine likely amphiphilic α-helical regions of the protein and the sites influenced by substrates or ligands. The hydrophobic moment, denoted as <*μ* H>, is a measure of amphiphilicity inside regular repeat structures that interprets the surface properties of amino acid residues as a two-dimensional vector sum (Phoenix and Harris, 2002).

Proteins carry out their biological functions by interacting directly with other molecules such as proteins and peptides, polynucleotides, membranes, receptors, and small molecule ligands including oxygen, solvent, and metal (Du et al., 2016). Addressing the factors and mechanisms that are responsible for protein–ligand interactions is very important for a better understanding of their effects on protein conformation and function under crowded conditions (Ellis, 2001, 2007; Goodsell, 2012; Du et al., 2016). Crowding agents (Ficoll and dextran) are highly soluble, inert, and do not intervene with spectroscopic experiments (Zimmerman and Minton, 1993). Their characteristic of being inert is crucial for associating theory and results since the excluded volume effect put emphasis on steric repulsions (Ellis, 2001; Minton, 2001). It is necessary to evade attractive interactions between the protein and the crowding agent of interest when studying volume exclusion. In contrast, PEG has been critiqued as not being an inert polymer and forms attractive interactions with protein along with the volume exclusion (Tubio et al., 2004; Bloustine et al., 2006; Winzor and Wills, 2006; Zhou et al., 2008). The PEGs used are not solid shapes, but rather flexible, soft, and permeable cloud-like structures. PEGs are polyether and have been suggested to have a mesh-like structure above the semi-dilute regime (Kozer et al., 2007; Crowley et al., 2008; Lee et al., 2008). PEG fractions with molecular weights greater than a few thousand have long been known to have a large and mostly repulsive interaction with proteins and to induce macromolecular associations and compaction in qualitative agreement with crowding theory (Jarvis et al., 1990; Reddy et al., 1995). Other water-soluble polymers and proteins (such as dextrans, Ficoll, haemoglobin, and bovine serum albumin or BSA) had been demonstrated to have less attractive interactions with other proteins, and their interactions with proteins can be predicted *via* excluded volume theories (Laurent, 1963; Rivas et al., 1999; Winzor and Wills, 2006). The above characteristics of PEGs may either stabilize or destabilize the proteins depending upon their size and shape, as in this study, the structure of the protein (cyt *c*) is seen to be perturbed in the presence of PEG 10 and more structured when the size of the crowder was increased doubly (PEG 20).

Earlier, we have studied the effects of various sizes of PEGs (PEG 400, PEG 10, and PEG 4000), their monomer (ethylene glycol, EG), and mixtures of crowders by varying their concentrations on the structural stability of heme-proteins (cyt *c* and myoglobin or Mb) (Parray et al., 2017; Parray et al., 2019; Parray et al., 2020a; Parray et al., 2020b; Parray et al., 2020c; Parray et al., 2021a; Parray et al., 2021b). The aim of these studies was to reflect on the updating progress being made through *in vitro* and *in silico* approaches to analyze the consequences of various sizes of macromolecular crowders on proteins and processes that stabilize the cell’s (*in vivo*) environment and to understand mechanisms of stabilization and destabilization due to various types of interactions in such conditions (Parray et al., 2019; Parray et al., 2020a; Parray et al., 2021a). Due to the presence of large number of macromolecules inside the cell, the effect of volume exclusion is particularly significant under *in vivo* and it takes place with all macromolecules (Chebotareva et al., 2004). The degree of volume exclusion between two molecules relies on their relative shapes and sizes. The species which are non-spherical can exhibit much larger excluded volumes than the spherical species of the same volume (Minton, 1981; Minton, 1998).

Our current study and the previous studies confirmed that the effect of the same crowder is different for different proteins (which means that size, shape, and structure of the protein play a great role in macromolecular crowding) and effect of different crowder molecules may be different for the same protein (which means that size, shape, and the structure of the crowder play a role in macromolecular crowding). The crowders may show either decrease or increase in the perturbation of the proteins’ characteristics depending on the type of interaction, whether the crowder interacts *via* soft interactions (destabilizing effect) or if it is excluded *via* exclusion volume effect (stabilizing effect) (Sarkar et al., 2013a; Sarkar et al., 2013b; Das and Sen, 2019; Nasreen et al., 2020; Parray et al., 2020a; Parray et al., 2021a). There are many studies which confirmed that small and large size of crowders show opposite effects on proteins, that is, small-sized crowders lead to destabilization and large-sized molecules lead to stabilization (Venturoli and Rippe, 2005; Rawat et al., 2010; Qu et al., 2012; Shahid et al., 2015; Sharp, 2015; Shahid et al., 2017; Biswas et al., 2018; Nasreen et al., 2018; Das and Sen, 2019; Shahid et al., 2019; Das and Sen, 2020; Parray et al., 2021a). Spectroscopic and binding studies by Rawat et al. (2010) suggested that a conformational change occurred in the protein (BSA) which was because of PEG interaction. They suggested behind this a molecular mechanism that leads PEG-mediated stabilization of the protein may be due to strong physical adsorption of PEG on the hydrophobic core of the protein along with surface adsorption led to the stability of protein (Rawat et al., 2010). The nuclear magnetic resonance (NMR)-based technique had also been used to know and understand the mechanism of interaction of PEG with cyt *c* (Crowley et al., 2008). Tokuriki et al. (2004) showed that large-sized crowders (35% of PEG 20 and Ficoll 70) leads to stabilization of the protein (ribonuclease A) using various spectroscopic techniques and NMR. From these studies, it can be suggested that macromolecules favored the compact conformations in the presence of high–molecular weight crowders at high concentrations, indicating the importance of a crowded environment for the folding and stabilization of globular proteins (Tokuriki et al., 2004).

Moreover, such reports suggest that in the medium (*in vitro*), two processes monitor structural changes of proteins simultaneously and can be mimicked with cellular conditions where the milieu is highly crowded and proteins are surrounded by various sizes and shapes of other macromolecules (Minton, 1981; Ellis, 2001; Goodsell, 2012; Sarkar et al., 2013b; Parray et al., 2020a; Parray et al., 2021b). In the case of PEGs, exclusion volume effect is not only the mechanism but soft interactions also play an important role in stabilizing intermediate states. Besides, the studies confirmed that PEGs are no more inert molecules and occasionally show strong binding (depending on the size of the PEG) with various proteins including heme-proteins from our study (Rawat et al., 2010; Qu et al., 2012; Sarkar et al., 2013a; Parray et al., 2019; Parray et al., 2020b; Parray et al., 2020d; Parray et al., 2021a). The effects of volume exclusion and soft interactions on the free energy of macromolecule in crowded and confined systems, as well as the consequences of crowding and confinement on structural stability and intra-molecular interactions, has been addressed. Overall, our finding suggests that proteins can be influenced in cellular crowded conditions *via* distinct routes and interactions, depending upon the size of the molecules surrounded.

## 5 Conclusion

PEG 10 kDa leads to destabilization of the protein’s tertiary structure with no significant effect on the secondary structure; on the contrary, the protein was more structured in the presence of PEG 20 kDa (double the size of PEG 10). Thus, different sizes of the crowder have different effects on the protein structure. The interaction studies (*in vitro* as well as *in silico*) showed that PEG 10 strongly binds *via* soft interactions (destabilizing effect) with the protein as compared to PEG 20 which showed exclusion volume effect, hence stabilizing the protein. This study confirms that protein structure and their functional activities are governed by both volume exclusion and soft (chemical) interactions. Comprehending the role of macromolecular crowding in the internal architecture of cellular environment and changes in the physicochemical characteristics of proteins, crowder size, and shape may have a significant role in proteinopathies in the cell.

## Data Availability

The original contributions presented in the study are included in the article/Supplementary Material, further inquiries can be directed to the corresponding author.
